# Raman hyperspectroscopy of saliva and machine learning for Sjögren’s disease diagnostics

**DOI:** 10.1038/s41598-024-59850-6

**Published:** 2024-05-15

**Authors:** Bhavik Vyas, Ana Khatiashvili, Lisa Galati, Khoa Ngo, Neil Gildener-Leapman, Melinda Larsen, Igor K. Lednev

**Affiliations:** 1 Department of Chemistry, University at Albany, SUNY, Albany, NY 12222 USA; 2 Division of Otolaryngology Head and Neck Surgery, Albany Medical College, Albany, NY 12208 USA; 3 Department of Biology and The RNA Institute, University at Albany, SUNY, Albany, NY 12222 USA

**Keywords:** Diagnostic markers, Immunological disorders, Oral diseases, Raman spectroscopy, Data processing, Learning algorithms

## Abstract

Sjögren's disease is an autoimmune disorder affecting exocrine glands, causing dry eyes and mouth and other morbidities. Polypharmacy or a history of radiation to the head and neck can also lead to dry mouth. Sjogren's disease is often underdiagnosed due to its non-specific symptoms, limited awareness among healthcare professionals, and the complexity of diagnostic criteria, limiting the ability to provide therapy early. Current diagnostic methods suffer from limitations including the variation in individuals, the absence of a single diagnostic marker, and the low sensitivity and specificity, high cost, complexity, and invasiveness of current procedures. Here we utilized Raman hyperspectroscopy combined with machine learning to develop a novel screening test for Sjögren's disease. The method effectively distinguished Sjögren's disease patients from healthy controls and radiation patients. This technique shows potential for development of a single non-invasive, efficient, rapid, and inexpensive medical screening test for Sjögren's disease using a Raman hyper-spectral signature.

## Introduction

Sjögren's syndrome disease (SjD) is a chronic autoimmune disorder characterized by salivary and lacrimal gland damage, mediated by the immune system, leading to eye and mouth dryness stemming from salivary gland and lacrimal gland hypofunction, respectively. SjD is a systemic disease that primarily affects the exocrine organs, can have pleomorphic clinical presentations, and as such, have a significant impact on a patient's quality of life. SjD can exist in a “primary” form if it is not associated with other diseases or “secondary” if it occurs concurrently with another autoimmune disorder such as Rheumatoid Arthritis^1^.

SjD affects middle-aged women significantly more than men, with the average female-to-male ratio being 9:1, irrespective of race and geographic location^2^. Although the diagnosis is often made later in life, with a mean age of 52–62 years^3^, the first symptoms may arise much earlier.

Like most autoimmune diseases, the exact etiology of SjD is unclear. Currently, the most widely accepted theory centers around exposure to environmental factors, especially viruses such as the Epstein–Barr virus^4^, which can cause dysregulation of the immune system.

The most common symptoms in SjD patients are ocular and mouth dryness^2^. Decreased saliva production often presents as dysphagia and dysgeusia, with difficulty swallowing dry foods and speaking for a prolonged period. Physical examination of patients with SjD typically demonstrates dry erythematous oral mucosa, often with dental caries or periodontal disease^5^. Chronic enlargement of a major salivary gland is also frequent^6^. In addition, low production of tears can lead to chronic ocular surface inflammation with signs such as photosensitivity, itching, and erythema^7^. Symptoms related to other gland dysfunctions, such as respiratory tract and skin dryness, can also occur in some patients^8^. These symptoms lead to a significant decline in quality of life for SjD patients.

Classification of SjD is complex and controversial. Although the American College of Rheumatology (ACR) and the European League Against Rheumatism (EULAR) have agreed on a set of criteria that were revised most recently in 2016^9^. The criteria are complex and require a score of 4 from 5 tests. Some of the diagnostic tools currently employed include the presence of antinuclear antibodies, including Ro/SSA and La/SSB antibodies^1^, but the presence of antibodies alone is insufficient to diagnose SjD, and not all patients have both antibodies. Other tests include an invasive salivary gland biopsy to identify focal lymphocytic sialadenitis and the presence of germinal centers^10^ and a measurement of salivary flow rate. In addition, patients are referred to an ophthalmologist to assess their lacrimal production via Schirmer's test and check the integrity of the epithelial layers of the cornea and conjunctiva via ocular staining^11^. No single evidence-based standardized screening test can diagnose patients who complain of dry mucous membranes. Because of the complexity of diagnosis and differing symptoms of patients, there is continued underdiagnosis of the disease^12^, limiting the ability to provide therapy early in the disease or even appropriately recruit patients to clinical trials.

Raman Spectroscopy (RS) of saliva has shown promising results in diagnosing various cancers, viral infections as well as autoimmune diseases like Alzheimer's disease^13–18^. Raman spectroscopy (RS) is a technique based on inelastic light scattering^19^, which probes the total (bio)chemical composition of the sample^20^. Recent scientific literature has demonstrated the potential of integrating Raman spectroscopy with machine learning techniques to distinguish individuals with Sjögren's disease from healthy individuals, utilizing human blood samples^21,22^. Saliva is an “ultra-filtrate” of blood and can reflect many pathological states^23^. Saliva collection is painless, non-invasive, and can be accomplished by the patient without a doctor’s visit. The ease of collecting saliva makes it possible to continue monitoring patients over time. Raman spectroscopy probes the total biochemical composition of a saliva sample.

However, the biochemical changes reflected as special variations on the Raman spectrum are often subtle and can be masked by instrumental drift and fluorescence background. The chemometrics techniques are being widely used to enhance the sensitivity of Raman spectroscopy for biological investigations, including data processing, data learning, and data interpretation. Machine learning techniques can achieve many chemometrics tasks, including classification and regression models^24^. Machine learning utilizes a complex Raman hyperspectral dataset to generate a spectral "fingerprint" of the disease, potentially including contributions from several biomarkers^25,26^.

In this proof-of-concept study, we demonstrated the potential of Raman hyperspectroscopy of saliva and machine learning for differentiating SjD patients from healthy control (HC) individuals and individuals treated with radiation therapy for head and neck cancers (RD), as these patients also suffer from salivary hypofunction and xerostomia. We demonstrate the effectiveness of using Raman hyperspectroscopy to differentiate between SjD, HC, and RD patients using a rapid, non-invasive saliva test.

## Results

Saliva samples (one sample per donor) were collected from 72 individuals representing HC, SjD, and RD at Albany Medical Center (AMC, Albany, NY) in accordance with the approved protocol of the AMC institutional review board (IRB). Nine randomly selected samples were set aside for external validation. The 63 remaining samples were used as a training dataset for a classification model. Thirty-six spectra were collected from each saliva sample using an automatic mapping technique.

Raman hyperspectroscopy takes advantage of a microheterogeneity of dry saliva to acquire information about various biochemical components, including those with a relatively low concentration, such as disease biomarkers^26^. Machine learning analysis of the Raman hyperspectral datacube (two spatial coordinates and a Raman spectrum) allows for developing a spectral signature of the disease, potentially including contributions from multiple biomarkers that can be used for disease diagnostics^26^.

**Figure 1:**
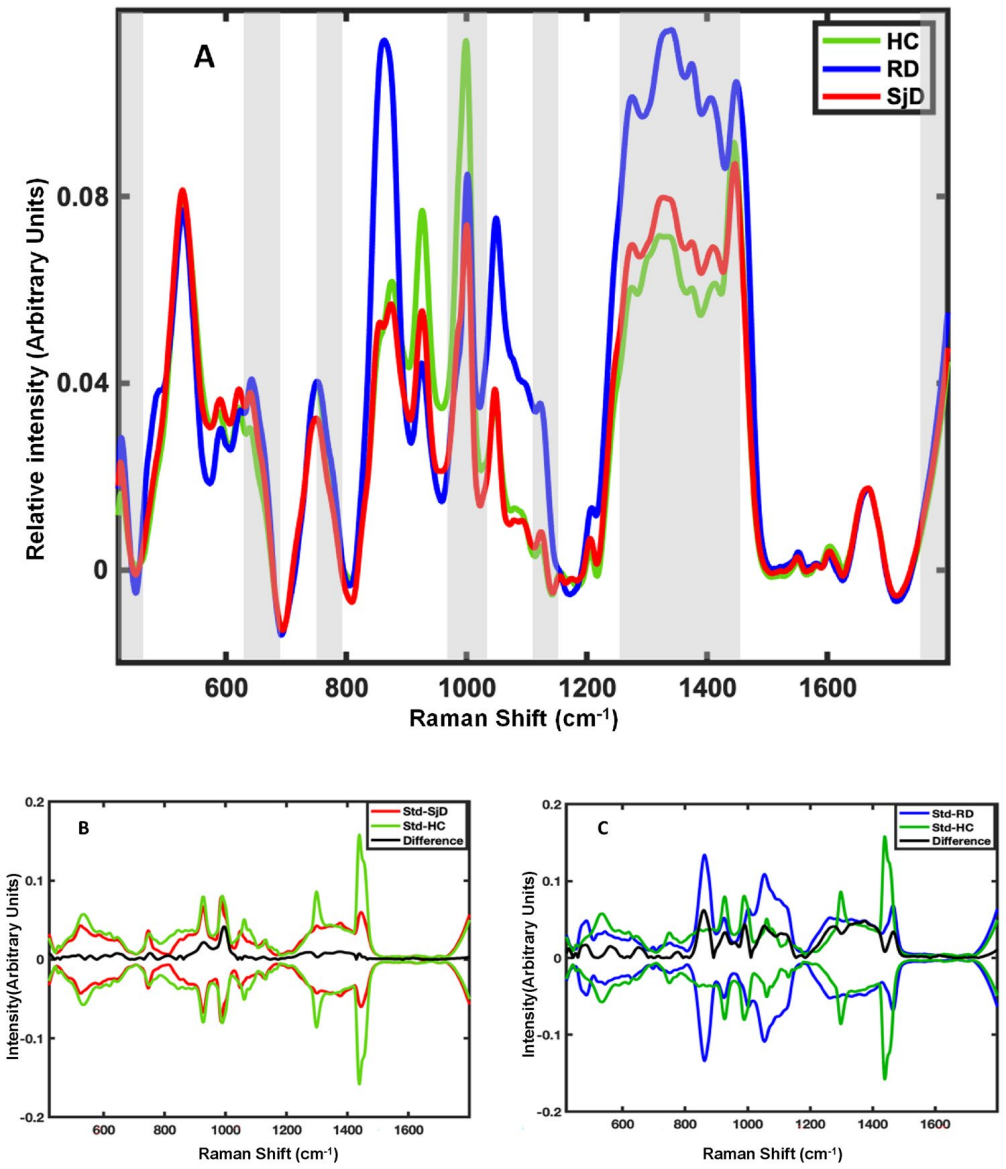
(**A**) Pre-processed mean Raman spectra of saliva acquired from Healthy controls (HC-green), Radiation therapy patients (RD-blue) and Sjögren's disease patients (SjD-red). Areas selected by Genetic Algorithm are highlighted (transparent grey). (**B**) A difference spectrum between SjD and HC mean spectra (black), and one standard spectral deviation of SjD (red) and HC (green) spectra. (**C**) A difference spectrum between mean spectra of RD and HC (black), and standard spectral deviation of RD (blue) and HC (green).

Mean preprocessed Raman spectra calculated for each class of donors, including HC, RD, and SjD, are shown in Fig. 1A. The spectra depict the biochemical composition of saliva with characteristic peaks and peak assignments based on literature, which is listed in Table 1. There are noticeable variations between the mean spectra in Fig. 1A. Yet, the difference spectrum between the SjD and HC mean spectra is within one standard spectral deviation of the SjD and HC classes (Fig. 1B). Similarly, the difference spectrum between RD and HC spectra remains within one standard deviation (Fig. 1C). The latter means that the variations between the mean spectra are statistically insignificant because the mean difference spectrum is well within the in-class standard spectral deviation of the groups intended. Given that the standard deviation exceeds the observed differences, it becomes evident that relying solely on one or two Raman bands' intensity values is insufficient for determining the healthy or diseased state. Instead, statistical analysis based on their entire spectra or significant parts is required^26,27^. This is not a surprising result as the biochemical composition of saliva might vary significantly because of environment, diet, and medical conditions, making spectral changes specific to the disease subtle. Supervised multivariate analysis, including machine learning algorithms, can identify these multiple small but specific differences between spectral signatures and build diagnostic classification models based on them.

**Table 1:** Tentative assignments of the main Raman bands of saliva based on literature data^13,43–46^.

Raman band (cm^−1^)	Tentative assignment
**426**	Proline (pyrrolidine ring deformation)*
528	S–S disulphide stretching band (collagen)
590	Glycerol, Cholesterol
622	Proteins (Phe), Lysozyme
640	Proteins (Tyr), Lysozyme
750	Proteins (Trp) Ring breathing mode
873	Proteins (Trp and Pro), Phosphatidylcholine
926	Proteins (Pro), glucose, Lactic acid
**1000**	Phe (Phenylalanine)*
**1048**	**CO**_**3**_^**–2**^, Phospholipids*
1122	Proteins (Trp), Lactic acid
**1154**	Phosphate present in DNA and RNA*
**1275**	C–C stretching mode of Proline(Pro)*
**1336**	**symmetric deformation vibration of the CH**_**3**_ group present in proteins, lipids, and other biomolecules*
**1373**	CH-deformation vibration of protein and lipids*
**1408**	**Symmetric bending vibration of CH**_**2**_ group of lipids*
1446	Deformation vibration of methyl group in lipids (–CH_3_)
1550	Proteins (Trp), Lysozyme
1602	Proteins (Phe and Tyr)
**1667**	Proteins (Amide I)*

Raman bands selected by Genetic Algorithm are highlighted with bold.

A type of supervised multivariate analysis, the Partial least squares-discriminant analysis (PLS_DA) model, was built to determine the number of latent variables and data distribution. Selecting an optimal number of latent variables improves the model's interpretability and reduces the risk of overfitting^28^. The PLS toolbox offers an outliers removal technique for the PLS_DA model called T^2^ Hotelling^29^. We used hotelling T^2^ scores with a conservative statistical threshold determined by PLS_DA to eliminate four outliers' samples, including 2-HC and 2-RD (Fig. S1, supplementary information). The final calibration dataset consisted of 59 donors with 1878 spectra and was introduced to GA (Genetic algorithm) for feature selection.

The Raman spectral dataset with many spectra per class is a high-dimensional dataset with various features^30^. Feature selection techniques like GA can reduce dimensionality by selecting only spectral components that show significant variations between classes of the dataset^31^. The operation of GA mimics Darwin’s rule of natural selection^32^. The objective is to pinpoint variables in the dataset that minimize the prediction error (Root Mean Square Error of cross-validation-RMSECV) for classification problems for the machine learning model through simulated natural selection, genetic mutations, and chromosome recombination^33^. In biological terms, natural selection embodies the notion of "survival of the fittest," wherein adaptation or evolution occurs via the elimination of weaker elements while optimal and sub-optimal elements are retained. Similarly, in GA, a problem solution is represented as a point in a search space termed a “chromosome," each encoding a combination of meaningful features^34^. Through exhaustive testing of potential solutions, GA generates populations of candidate solutions, ranking them based on a fitness function. The algorithm then applies operators such as crossover, mutation, inversion, and recombination to selected portions of the most promising solutions. This iterative computational process mimics natural reproduction, allowing only the most fit populations to reproduce until satisfactory results are achieved. GA excels in handling large search spaces, making it particularly suitable for scenarios involving spectral data with hundreds or thousands of variables.

Further, we employed the advanced machine learning classification technique Support vector machine-discriminant analysis (SVM_DA) to analyze collected spectral data for inter-class differences. With the help of GA, SVM_DA selects the area (data points) of the spectra specific to each class and generates a hyperplane (separating line) between classes for classification. Tentative assignments of important Raman bands selected by GA are available in Table 1 (highlighted in bold). The GA has selected bands assigned to Proline (426 cm^−1^, 1275 cm^−1^), phenylalanine (1000 cm^−1^), tryptophan (1048 cm^−1^, 1373 cm^−1^), and 1154 cm^−1^, 1336 cm^−1^, 1408 cm^−1^,1667 cm^−1^ that can be assigned to carotenoids, proteins and lipid. The average spectrum suggests that these bands are lower in intensity for SjD than those in HC and RD. This suggests a potential metabolic shift in SjD patients, leading to reduced levels of proline, carotenoids, and tryptophan compared to healthy individuals. Notably, previous studies of blood have demonstrated significant alterations in proline and tryptophan metabolic levels associated with the effects of SjD^35^, further supporting the importance of these findings in our study.

We imported the calibration spectral dataset created by GA into the SVM_DA model for training consisting of 1878 total spectra labeled with their respective classes. We used 11 LVs selected using PLS_DA to train an SVM_DA classification model. Next, we applied the custom cross-validation with 50 splits and approximately 20 spectra in each division. The latter means that the data was divided into 50 subsets of 20 spectra each for cross-validation. One subset at a time was left as a test for the model built based on the rest of the spectra. As a result, multiple SVM_DA models were trained based on different subsets of the data to evaluate the model’s robustness and generalizability. The cross-validation method applied here was analogous to k-fold cross-validation and, as such, indicated that the built SVM_DA model is not overfitted^36^. The confusion matrix for the built SVM_DA model’s cross-validation prediction at the spectral level can be found in Table 2. The SVM-DA model offered cross-validation sensitivity (true positive rate) of 86% for SjD (Table 3) at a spectral level. We collected 36 spectra per sample to represent sample heterogeneity. A 97% accuracy at the sample level (2 samples from 63 were misclassified) was achieved by SVM_DA using a 50% threshold since the majority of spectra were correctly assigned to their actual class.

**Table 2:** Cross-validation predictions for individual spectra collected for samples in the calibration dataset.

Predicted class	Actual class		
	HC	RD	SjD
HC	494	25	69
RD	23	440	36
SjD	76	57	658

**Table 3:** The performance matrix of SVM_DA cross validation at spectral level.

Cross validation	HC	RD	SjD
Sensitivity (true positive rate)	0.83	0.84	0.86
Specificity (true negative rate)	0.93	0.96	0.88
Class. Error (miss classification)	0.12	0.10	0.13

**Figure 2:**
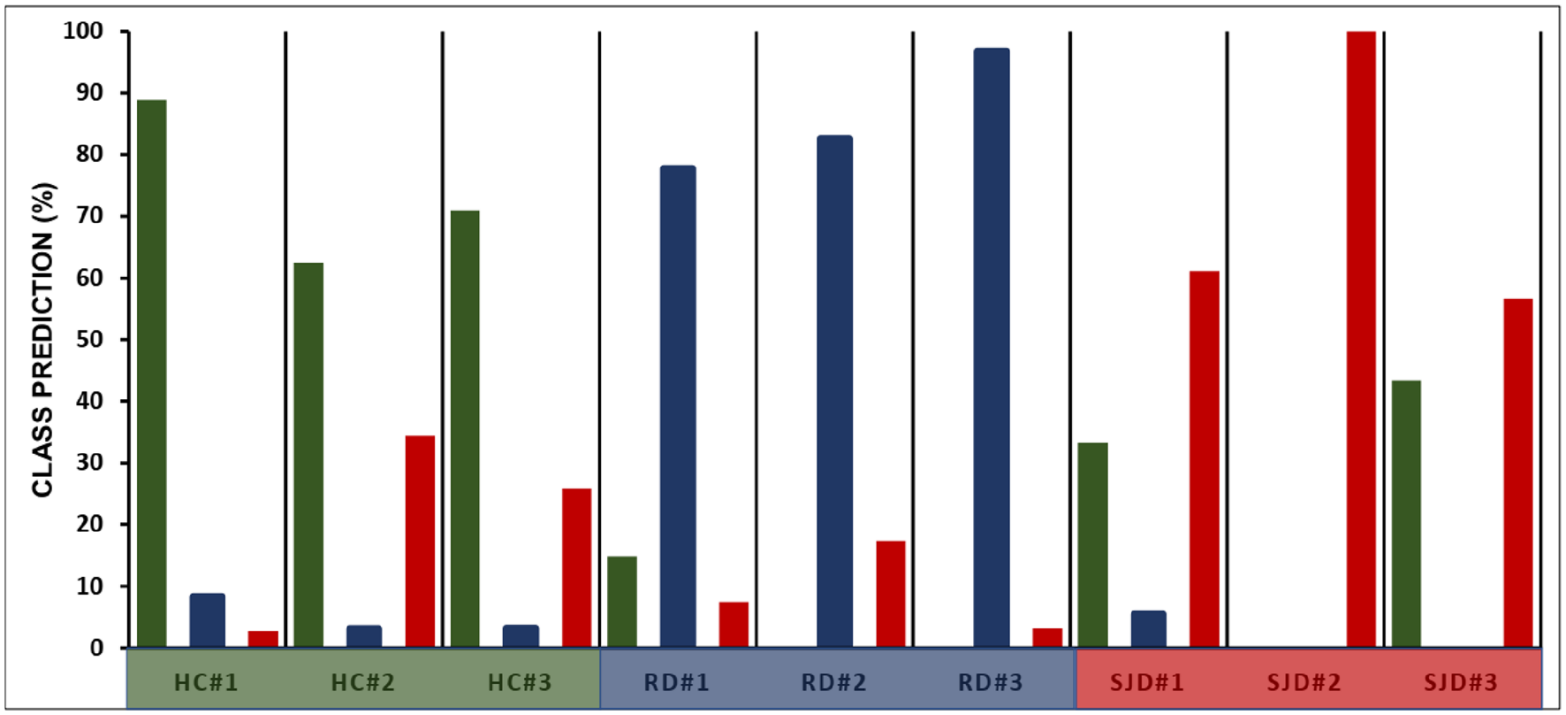
External validation of the SVM_DA model. The percent spectra assigned to HC (green), RD (blue), and SjD (red) classes are reported for individual samples.

The ultimate test for the validity of a classification model is its external validation based on samples not included in the training dataset. We performed the external validation of the SVM_DA model using nine samples not used to create the model. In order to divide the dataset for calibration and external validation purposes, we opted for a random selection method without any criteria. The random selection ensures an unbiased distribution of samples and does not limit any sample to be in the external validation dataset. Further, the model’s performance was evaluated primarily based on cross-validation techniques. This deliberate choice aimed to maintain the external validation dataset’s anonymity to the model, ensuring an unbiased assessment of its true potential and mitigating any inherent biases. The efficacy demonstrated through this rigorous evaluation process underscores the model’s reliability and generalizability. The confusion matrix revealed that an SVM_DA model showed 79% accuracy at the spectral level, with some spectra assigned to incorrect classes (Table S1A, supplementary information SI). The prediction of nine external validation samples is summarized in Fig. 2 and Table S1B (SI). The histogram shows that all nine samples were assigned to their actual class by the SVM_DA model at the 50% threshold. Moreover, the model can not only successfully differentiate between Sjögren disease patient saliva and healthy saliva but also distinguish between radiation therapy patient saliva and Sjögren disease patient saliva.

## Discussion

This proof-of-concept study demonstrated that Raman hyperspectroscopy combined with machine learning can successfully differentiate patients with Sjögren’s disease from head and neck cancer radiation patients and healthy individuals on the basis of a non-invasive saliva test. While the investigation was carried out utilizing a constrained sample size of 72 patients, it is noteworthy that accurate predictions were achieved across all nine external validation samples. The *P* value (*p* > 0.05, Table 4) indicates that gender cannot be a significant factor for the classification as the number of male and female samples is sufficient to support the null hypothesis. This outcome underscores the robustness of the developed model, indicating its impartiality towards gender and its reliance on spectroscopic markers indicative of healthy or disease states.

**Table 4:** Information about the donors’ age and sex for Healthy control (HC), Radiation (RD), and Sjögren's disease patients (SjD).

	HC (n = 22)	RD (n = 22)	SjD (n = 26)	Significant *p* value
Age	60 (± 10)	66 (± 9)	60 (± 10)	> 0.05
Sex				> 0.05
Male	14	18	4	
Female	8	4	22	

This proof-of-concept study was based on a limited age range of 62 ± 10 years, aligning with the higher prevalence of the disease among middle-aged women. The P-value supports that gender cannot be a significant factor for the classification. Moving forward, our research endeavors will focus on expanding our cohort's size and diversity. Broadening the donor’s population will allow to capture a more comprehensive representation of the demographic variability associated with the disease.

Raman spectra were collected from multiple spots on heterogeneous dry saliva samples to increase the probability of detecting specific disease biomarkers, which are typically present at a low concentration. A single reading from the sample is insufficient, and multiple readings and full spectral-level predictions are required to make the final classification at the donor level. In our earlier study, the development of Alzheimer’s disease from the mild to moderate stage increased the number of specific disease biomarkers in blood and, as a result, significantly increased the portion of individual Raman spectra in the hyperspectral datacube, which was characteristic of the disease^26^.

Raman hyperspectroscopy is ideal for disease diagnostic tests due to its non-invasive nature, rapid analysis, and high sensitivity in detecting molecular changes associated with various diseases. The abundance of biomolecules in saliva allows for the identification of potential disease biomarkers, while the cost-effectiveness and portability of Raman spectroscopy make it feasible for point-of-care applications and resource-limited settings, enabling early disease detection and monitoring^37^.

Raman hyperspectroscopy of saliva holds great promise for the development of a non-invasive, efficient, rapid, and inexpensive diagnostic test for SjD. Due to the non-invasive nature of the test, it could be used to screen patients for participation in clinical trials and follow disease progression or response to treatment. It might also be useful to identify early stages of disease development; however, further work will be required to determine at what stage the disease can be detected with Raman hyperspectroscopy. Although we demonstrated the ability to distinguish between SjD and radiation-induced xerostomia, testing more samples is required to validate the developed model's sensitivity and selectivity further relative to other diseases.

This method could have broader applicability for those patients with radiation to the head and neck. For these patients, spectral analysis should be correlated with specific radiation doses and used to track saliva quality over time. Another important research direction is to examine the potential effect of medication regimens on the Raman signature and to characterize patients with xerostomia secondary to polypharmacy, which could also have clinical utility for patient monitoring.

## Material and methods

### Saliva samples

Saliva samples were collected from 72 donors (one sample per donor, 24-HC, 26-SjD, 22-RD) at Albany College of Medicine under the approval of the Institutional Review Board (IRB) and stored at -20C. The Age information about the donors can be found in Table 4. Participants were refrained from food, beverages, chewed gum, or smoked 30 min before sample collection. The oral radiation population consisted of individuals who had completed previous oral radiation therapy, were currently in remission from cancer, and were experiencing xerostomia as a resultant condition. Patients diagnosed with Sjögren’s disease were characterized by rheumatologists, presently undergoing treatment, reporting xerostomia, displaying multiorgan involvement, and testing positive for Anti-SSA/Anti-SSB or at least one of the Early Sjögren’s disease Antibodies. The control group comprised individuals without oral dryness symptoms, no identifiable oral health concerns, and no history of oral cancer. Samples were thawed and centrifuged for 5 min at 20,000 rpm. The supernatant was collected and used for the Raman spectral analysis. About 10 μL of saliva supernatant was deposited on an aluminum foil-covered glass slide and dried overnight. Drying saliva samples allows for leveraging its heterogeneous nature, enabling the extraction of information regarding its individual components and their alterations associated with the disease^38^. The aluminum foil minimizes substrate interference^39^.

### Ethics approval and accordance

This study was approved by the Institutional Review Board (IRB) at Albany College of Medicine. Informed consent was obtained from all participants and/or their legal guardians before participating in the study. All methods were carried out in accordance with relevant guidelines and regulations provided by the IRB (e.g., The Belmont Report).

### Raman hyperspectroscopy

All Raman spectra were collected using a Horiba Xplora-Plus Raman microscope (HORIBA Scientific). The PRIOR automatic mapping stage was used to collect Raman spectra from multiple locations on dry saliva samples using a 50X objective to probe the sample heterogeneity and generate the hyperspectral datacube^40,41^. Spectra were recorded in the range of 400–1800 cm^-1^ using a 785-nm laser source with 100% power (110mW). A total of 36 spectra per sample were collected with a 30-s acquisition time at each location and three accumulations at each location using LabSpec6 software (Version 6.1, software available at https://www.horiba.com/usa/scientific/products/detail/action/show/Product/labspec-6-spectroscopy-suite-software-1843/). The PRIOR automatic stage moves the sample stage to each designated point according to a predefined grid and autofocuses for spectral acquisition. The movement is precise and automated, eliminating the need for manual intervention to collect multiple spectra in a grid-like fashion.

### Data analysis

A total of 2264 spectra from 72 saliva samples were imported into MATLAB (R2019b) programming software (MathWorks, Inc) equipped with PLS-Toolbox 9.0 (2021) (Eigenvector Research, Inc., Manson, WA USA 98831; software available at http://www.eigenvector.com). Raman spectra with extensive cosmic rays or low signal-to-noise ratio were removed from the dataset. Further, the automatic preprocessing was applied to all spectra in the training dataset using the PLS Toolbox, including first baseline correction (weighted least square, order 6), then normalization by 1667-cm^−1^ band, and at last smoothing (Sav Gol filter width 31, order 5)^42^. This band (1667-cm^−1^), tentatively assigned to protein Amid I vibrational mode, showed the least variations among strong Raman bands in saliva spectra. Nine samples (3-HC,3-SjD,3-RD) were randomly selected and set aside for external validation. We assigned classes to each spectrum in the training dataset. Next, we applied feature selection techniques, such as the GA, to select spectral components from the training dataset. The parameters of GA are given as follows: the population size was set to 62, the mutation rate to 0.005, and the maximum number of generations for every run was set to 100. We used double cross-over breeding with a window width of 30%. We ran GA 100 times independently to select diagnostic feature information from the measured Raman spectra of the calibration dataset. Furthermore, standard hyperparameters offered by the PLS_Toolbox were utilized to construct the SVM_DA model, incorporating GA-selected variables from the training dataset. The model was configured with the RBF kernel function and PLS compression with a compression component of 11 (compressncomp 11). These default parameters are optimized for general use across various datasets, balancing model complexity and performance. The software version used was PLS_Toolbox 9.0 from Eigenvector Research, Inc. (Manson, WA, USA, 98831). Once the model's performance was optimized, an external validation dataset was introduced to the built SVM_DA model, following the same preprocessing steps as the training dataset. The cross-validation and external validation were performed to test the model’s performance.

### Supplementary Information

Supplementary Information.

## Data Availability

The datasets used and analyzed during the current study are available from the corresponding author on reasonable request.
