# Supplementary material for: Raman hyperspectroscopy of saliva and machine learning for Sjögren’s disease diagnostics
Source: Sci Rep. 2024 May 15;14:11135. doi: 10.1038/s41598-024-59850-6 (PMC11096345; doi:10.1038/s41598-024-59850-6)
Supplement: Supplementary file 1 — Supplementary Information. [file 41598_2024_59850_MOESM1_ESM.pdf]

Supplementary Information

Raman hyperspectroscopy of saliva and machine learning for Sjögren's Syndrome diagnostics

Bhavik Vyas[1], Ana Khatiashvili[2], Lisa Galati[2], Khoa Ngo[2], Neil Gildener-Leapman[2], Melinda Larsen[3] and Igor K. Lednev[1,3*]

[1]*Department of Chemistry, University at Albany, SUNY, Albany, NY 12222*

[2]*Division of Otolaryngology Head and Neck Surgery, Albany Medical College, Albany NY 12208*

[3]*Department of Biology and The RNA Institute, University at Albany, SUNY, Albany, NY 12222*

[*]*Corresponding Author: ilednev@albany.edu*

Fig S1. Outlier removal using Hoteling T2 . The spectra with the T2 Hotteling values exceeding reduced statistical threshold (--) considered outliers (Grey area top right)

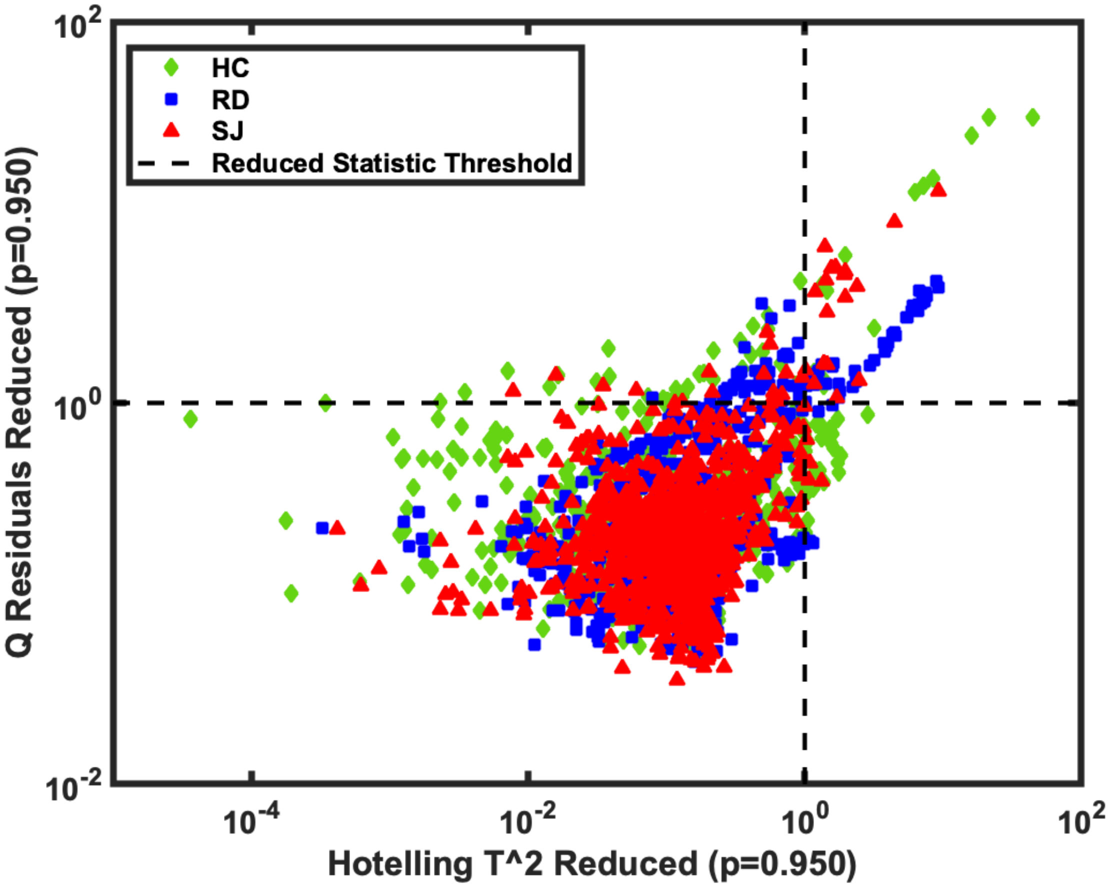

Table S1A. Confusion matrix of the external validation at the spectral level

Predicted Class	Actual Class		
	HC	RD	SJ
HC	74	4	19
RD	5	70	1
SJ	20	7	65

Table S1B. Classification predictions for individual spectra collected for each of the nine samples used for external validation.

True class	Predicted class		
	HC	RD	SjD
HC-1	32	3	1
HC-2	20	1	11
HC-3	22	1	8
RD-1	4	21	2
RD-2	0	19	4
RD-3	0	30	1
SjD-1	6	1	11
SjD-2	0	0	37
SjD-3	13	0	17
